# Supplementary material for: Resolving the full spectrum of human genome variation using Linked-Reads
Source: Genome Res. 2019 Apr;29(4):635–45. doi: 10.1101/gr.234443.118 (PMC6442396; doi:10.1101/gr.234443.118)
Supplement: Supplemental Material [file supp_gr.234443.118_Supplemental_longranger-2.2.zip › longranger-2.2.tar.bz2!/lr2.2-public/lib/stan/cmdstan/stan/doc/api/html/math_2matrix_2quad__form_8hpp_source.html]

Stan: src/stan/math/matrix/quad\_form.hpp Source File


|  |  |
| --- | --- |
|  | Stan  2.5.0  probability, sampling & optimization |


- Main Page
- Related Pages
- Namespaces
- Classes
- Files

- File List
- File Members

All Classes Namespaces Files Functions Variables Typedefs Enumerations Enumerator Friends Macros Pages

- src
- stan
- math
- matrix

quad\_form.hpp

Go to the documentation of this file.

1 #ifndef STAN\_\_MATH\_\_MATRIX\_\_QUAD\_FORM\_HPP

2 #define STAN\_\_MATH\_\_MATRIX\_\_QUAD\_FORM\_HPP

3

4 #include <boost/utility/enable\_if.hpp>

5 #include <boost/type\_traits.hpp>

6 #include <stan/math/matrix/Eigen.hpp>

7 #include <stan/math/error\_handling/matrix/check\_multiplicable.hpp>

8 #include <stan/math/error\_handling/matrix/check\_square.hpp>

9 #include <stan/math/error\_handling/matrix/check\_symmetric.hpp>

10 #include <stan/math/matrix/dot\_product.hpp>

11 #include <stan/math/matrix/multiply.hpp>

12 #include <stan/math/matrix/transpose.hpp>

13

14 namespace stan {

15  namespace math {

19  template<int RA,int CA,int RB,int CB,typename T>

20  inline Eigen::Matrix<T,CB,CB>

21  quad\_form(const Eigen::Matrix<T,RA,CA>& A,

22  const Eigen::Matrix<T,RB,CB>& B)

23  {

24  using stan::math::multiply;

25  stan::math::check\_square("quad\_form(%1%)",A,"A",(double\*)0);

26  stan::math::check\_multiplicable("quad\_form(%1%)",A,"A",

27  B,"B",(double\*)0);

28  return multiply(stan::math::transpose(B),multiply(A,B));

29  }

30

31  template<int RA,int CA,int RB,typename T>

32  inline T

33  quad\_form(const Eigen::Matrix<T,RA,CA>& A,

34  const Eigen::Matrix<T,RB,1>& B)

35  {

36  using stan::math::multiply;

37  using stan::math::dot\_product;

38

39  stan::math::check\_square("quad\_form(%1%)",A,"A",(double\*)0);

40  stan::math::check\_multiplicable("quad\_form(%1%)",A,"A",

41  B,"B",(double\*)0);

42  return dot\_product(B,multiply(A,B));

43  }

44

45  template<int RA,int CA,int RB,int CB,typename T>

46  inline Eigen::Matrix<T,CB,CB>

47  quad\_form\_sym(const Eigen::Matrix<T,RA,CA>& A,

48  const Eigen::Matrix<T,RB,CB>& B)

49  {

50  using stan::math::multiply;

51

52  stan::math::check\_square("quad\_form\_sym(%1%)",A,"A",(double\*)0);

53  stan::math::check\_multiplicable("quad\_form\_sym(%1%)",A,"A",

54  B,"B",(double\*)0);

55  stan::math::check\_symmetric("quad\_form\_sym(%1%)",A,"A",(double\*)0);

56  Eigen::Matrix<T,CB,CB> ret(multiply(stan::math::transpose(B),multiply(A,B)));

57  return 0.5\*(ret + stan::math::transpose(ret));

58  }

59

60  template<int RA,int CA,int RB,typename T>

61  inline T

62  quad\_form\_sym(const Eigen::Matrix<T,RA,CA>& A,

63  const Eigen::Matrix<T,RB,1>& B)

64  {

65  using stan::math::multiply;

66  using stan::math::dot\_product;

67

68  stan::math::check\_square("quad\_form\_sym(%1%)",A,"A",(double\*)0);

69  stan::math::check\_multiplicable("quad\_form\_sym(%1%)",A,"A",

70  B,"B",(double\*)0);

71  stan::math::check\_symmetric("quad\_form\_sym(%1%)",A,"A",(double\*)0);

72  return dot\_product(B,multiply(A,B));

73  }

74  }

75 }

76

77 #endif

78

check\_multiplicable.hpp

stan::math::quad\_form\_sym

Eigen::Matrix< T, CB, CB > quad\_form\_sym(const Eigen::Matrix< T, RA, CA > &A, const Eigen::Matrix< T, RB, CB > &B)

**Definition:** quad\_form.hpp:47

check\_symmetric.hpp

transpose.hpp

stan::math::check\_symmetric

bool check\_symmetric(const char \*function, const Eigen::Matrix< T\_y, Eigen::Dynamic, Eigen::Dynamic > &y, const char \*name, T\_result \*result)

Return true if the specified matrix is symmetric.

**Definition:** check\_symmetric.hpp:31

stan::math::check\_multiplicable

bool check\_multiplicable(const char \*function, const T1 &y1, const char \*name1, const T2 &y2, const char \*name2, T\_result \*result)

**Definition:** check\_multiplicable.hpp:15

stan::math::transpose

Eigen::Matrix< T, C, R > transpose(const Eigen::Matrix< T, R, C > &m)

**Definition:** transpose.hpp:12

Eigen.hpp

dot\_product.hpp

stan::math::multiply

boost::enable\_if\_c< boost::is\_arithmetic< T >::value, Eigen::Matrix< double, R, C > >::type multiply(const Eigen::Matrix< double, R, C > &m, T c)

Return specified matrix multiplied by specified scalar.

**Definition:** multiply.hpp:25

stan::math::quad\_form

Eigen::Matrix< T, CB, CB > quad\_form(const Eigen::Matrix< T, RA, CA > &A, const Eigen::Matrix< T, RB, CB > &B)

Compute B^T A B.

**Definition:** quad\_form.hpp:21

multiply.hpp

stan::math::check\_square

bool check\_square(const char \*function, const Eigen::Matrix< T\_y, Eigen::Dynamic, Eigen::Dynamic > &y, const char \*name, T\_result \*result)

Return true if the specified matrix is square.

**Definition:** check\_square.hpp:24

stan::math::dot\_product

double dot\_product(const Eigen::Matrix< double, R1, C1 > &v1, const Eigen::Matrix< double, R2, C2 > &v2)

Returns the dot product of the specified vectors.

**Definition:** dot\_product.hpp:22

check\_square.hpp


---

[ Stan Home Page ]

*© 2011–2014,
Stan Development Team.*
